# Supplementary material for: Predicting visual function by interpreting a neuronal wiring diagram
Source: Nature. 2024 Oct 2;634(8032):113–23. doi: 10.1038/s41586-024-07953-5 (PMC11446822; doi:10.1038/s41586-024-07953-5)
Supplement: Supplementary file 6 — CRF and ERF predictions for individual TmY4 and TmY9 cells. Analogous to Supplementary Data 3, but for TmY target types. Shown are the top four monosynaptic pathways, the strongest pathway passing through each of the top ten intermediary types (ranking from Extended Data Fig. 7), and the trisynaptic pathway Tm1–TmY–Dm3–TmY (see the section entitled Prediction of spatial normalization). [file 41586_2024_7953_MOESM6_ESM.zip › DataS4/TmY9qΓèÑ/720575940615234410.pdf]

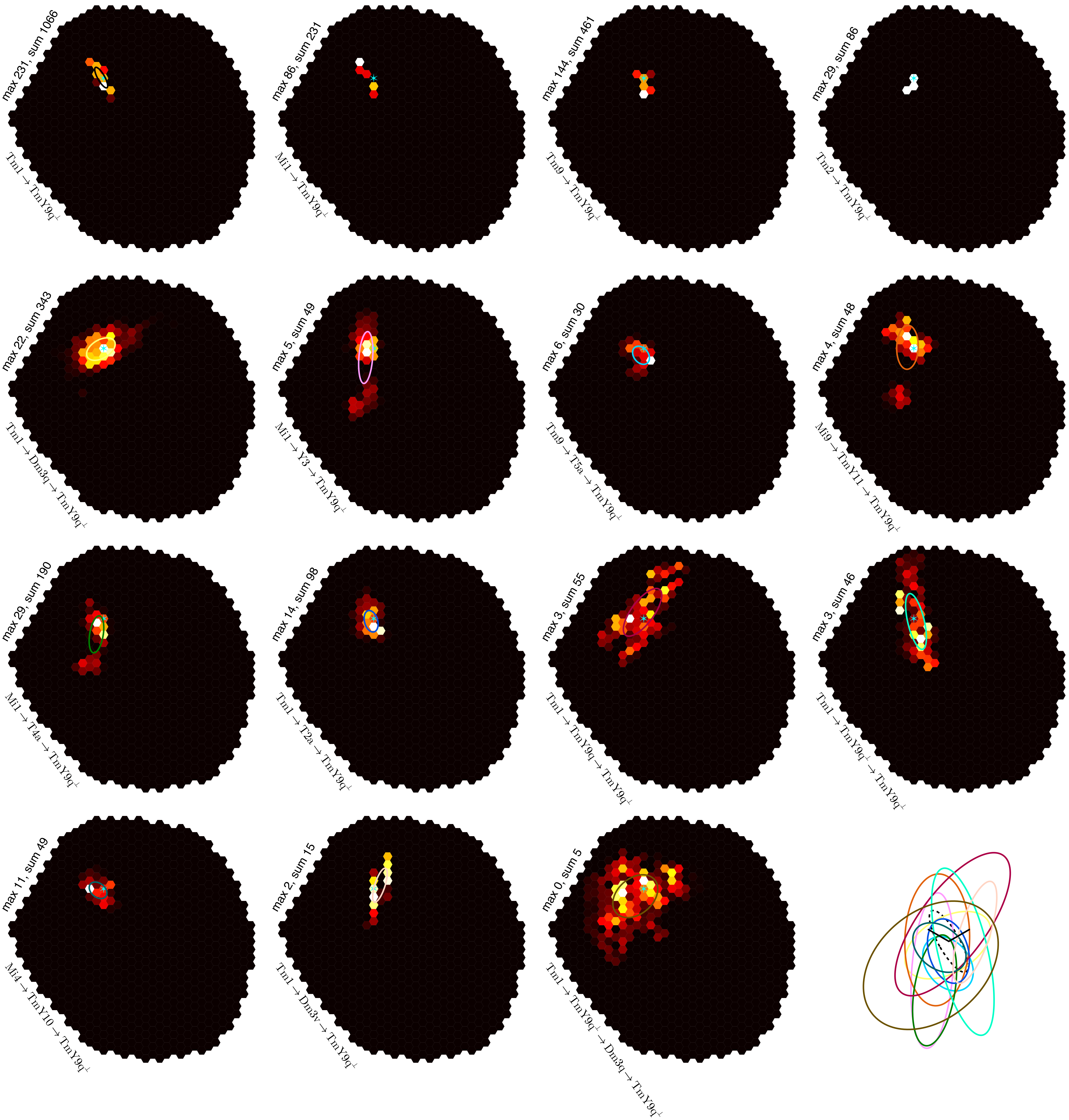
max 231, sum 1066
Tm1 → TmY9q⊥
max 86, sum 231
Mi1 → TmY9q⊥
max 144, sum 461
Tm9 → TmY9q⊥
max 29, sum 86
Tm2 → TmY9q⊥
max 22, sum 343
Tm1 → Dm3q → TmY9q⊥
max 5, sum 49
Mi1 → Y3 → TmY9q⊥
max 6, sum 30
Tm9 → T5a → TmY9q⊥
max 4, sum 48
Mi9 → TmY11 → TmY9q⊥
max 29, sum 190
Mi1 → T4a → TmY9q⊥
max 14, sum 98
Tm1 → T2a → TmY9q⊥
max 3, sum 55
Tm1 → TmY9q → TmY9q⊥
max 3, sum 46
Tm1 → TmY9q⊥ → TmY9q⊥
max 11, sum 49
Mi4 → TmY10 → TmY9q⊥
max 2, sum 15
Tm1 → Dm3v → TmY9q⊥
max 0, sum 5
Tm1 → TmY9q⊥ → Dm3q → TmY9q⊥
